# The Child Affective Facial Expression Set Short Versions (CAFE-Ss): Development and Validation of Two Subsets of Children’s Emotional Faces With Variability

**DOI:** 10.3389/fpsyg.2020.599245

**Published:** 2020-12-04

**Authors:** Yang Yang, Vanessa LoBue

**Affiliations:** ^1^National Institute of Education, Nanyang Technological University, Singapore, Singapore; ^2^Department of Psychology, Rutgers University, Newark, NJ, United States

**Keywords:** face, emotion expressions, CAFE, stimulus sets, child

## Abstract

Emotion recognition plays an important role in children’s socio-emotional development. Research on children’s emotion recognition has heavily relied on stimulus sets of photos of adults posed stereotyped facial configurations. The Child Affective Facial Expression set (CAFE) is a relatively new stimulus set that provides researchers with photographs of a diverse group of children’s facial configurations in seven emotional categories—angry, sad, happy, fearful, disgusted, surprised, and neutral. However, the large size of the full CAFE set makes it less ideal for research in children. Here, we introduce two subsets of CAFE with 140 photographs of children’s facial configurations in each set, diverse in the race and ethnicity of the models, and designed to produce variability in naïve observers. The subsets have been validated with 1000 adult participants.

## Introduction

Ekman and colleagues classically argued that there are a limited set of basic emotions—including happiness, sadness, anger, fear, disgust, and surprise—that can be recognized universally and from an early age (e.g., Ekman and Friesen, 1971). Based on the assumption that stereotypical facial configurations that represent these basic emotions are universally expressed and recognized, previous research on emotion recognition has overwhelmingly depended on photographs of adults posing stereotypical configurations of these basic emotion categories. While such stimulus sets provide an easy and controlled way of examining responses to human facial expressions, they come with several important limitations.

First and foremost, most available stimulus sets of emotional facial configurations only capture the faces of one particular demographic—namely, Caucasian adults, with a few exceptions (e.g., Chicago Face Database, Ma et al., 2015). These sets generally contain very little racial and ethnic diversity among exemplars, and photographic sets of children are extremely rare. This is problematic, as individual differences in emotion recognition have been widely reported. For example, people tend to better recognize emotional expressions displayed by members from their own group (race, country, region, or ethnicity) when compared to those from another group (Elfenbein and Ambady, 2002). Further, several studies have shown substantial cross-cultural variation in the ways people express different emotions. For example, while Westerners display some stereotypical emotional expressions with a distinct set of facial muscles, East Asians demonstrate considerable overlap between expressions, particularly for surprise, fear, disgust, and anger (Jack et al., 2012). Further, while adults from Western countries rely heavily on verbal content and facial information to make inferences about emotion categories, adults from Japan rely more on contextual information, such as voices (Ishii et al., 2003; Tanaka et al., 2010). This suggests that using exemplars posed by a single demographic can ignore significant variability in the way that different individuals might express different emotions.

Second, most stimulus sets typically contain only highly stereotyped, high-intensity exemplars. Not surprisingly, these facial configurations are generally identified with a high degree of accuracy among naïve adults and even young children. This is also problematic, as people express emotions with considerably more variation than is represented in these posed stereotypical, static configurations (Barrett et al., 2019). Moreover, a given facial configuration (e.g., scowling) can represent various emotion categories (e.g., anger and sadness). Indeed, research on children’s emotion recognition has shown that when the photographs of posed configurations are more variable, children’s accuracy improves gradually over time, and only becomes comparable to adults’ around the age of 10 or older (Gao and Maurer, 2010).

To address these issues for research on emotional development, LoBue and Thrasher (2015) created the Child Affective Facial Expression Set (CAFE), which contains photographs of young children (2–8 years old) from a diverse population posing the six basic emotions—sadness, happiness, surprise, anger, disgust, and fear—plus a neutral expression (LoBue and Thrasher, 2015). CAFE is ethnically and racially diverse, featuring Caucasian, African American, Latino (Hispanic), Asian, and South Asian (Indian/Bangladeshi/Pakistani) children, allowing researchers to study children from diverse racial or ethnic backgrounds on recognizing emotional expressions displayed by children in their own and other races/ethnicities. Further CAFE contains a large number of exemplars (∼1200) with a wide amount of variability in how much each exemplar resembles a stereotypical emotional expression and how difficult the emotional expression can be recognized by a naïve observer. In fact, CAFE includes one subset of faces (Subset A) that contains only highly stereotypical exemplars of the various emotion categories, consistent with other existing face sets, and a second subset (Subset B) that only includes faces that emphasize variation around emotion targets in research participants while minimizing potential ceiling and floor effects. This variability better enables researchers to study the natural variation in human emotional expressions and allows researchers to study individual differences in people’s ability to recognize emotional expressions. However, the large size of the CAFE set can make it challenging to use, particularly in studies with children. For example, most studies designed for children could not possibly make use of 1200 exemplars, forcing researchers to hand pick a small subset of faces. However, CAFE is only optimized for capturing natural variability in a sample of naïve observers when the *whole set is used*.

Here, we aimed to develop and validate a new subset of CAFE that contains photographs of a diverse group of children and is optimized for capturing variability in a sample of naïve observers, but that only contains less than 150 exemplars^1^. In Study 1, we used data from LoBue and Thrasher (2015) to select CAFE faces that vary in how difficult they are to identify by naïve observers, and in Study 2, we pilot-tested the selected faces with a sample of 100 adult participants. In Study 3, we validated the new subset with a larger sample (*N* = 1000), calculated accuracy scores, and ensured that these scores were balanced between genders and races/ethnicities of the faces. Information on the new validated subset(s) is available for use by the scientific community on Databrary^2^.

## Study 1

The purpose of Study 1 was to identify small subsets of photographs from CAFE that resemble the full set in composition. Like the full CAFE set, these subsets would be ideal for studying individual differences in recognizing emotional expressions, but contain a much smaller set size (∼140 faces), making them more appropriate for studies with children.

### Method

#### Procedure

The CAFE set is a validated stimulus set of 2–8-year-old children (*M* = 5.3 years; *Range* = 2.7–8.7 years) posing for six emotional facial expressions—sadness, happiness, surprise, anger, disgust, and fear—plus neutral (LoBue and Thrasher, 2015). We selected our small subsets from the photographs in Subset B of CAFE^3^. Subset B contains a selection of faces from the full set (1090 of the full 1192 items) that emphasize variation around emotion targets in research participants while minimizing potential ceiling and floor effects (as identified by latent response models). The faces in Subset B were identified by first asking 100 naïve adult raters to identify the emotion categories posed by each of the faces in the entire CAFE set, and then by calculating an accuracy score for each face that represented the proportion of 100 adult raters who identified the face correctly, ranging from 0 (0% of participants) to 1 (100% of participants) (LoBue and Thrasher, 2015). A one-parameter logistic or *Rasch model* was then applied to this data to calculate a standardized difficulty score (*b*_*i*_), along with fit statistics (in-fit and out-fit), for each face photograph in the set, in order to indicate whether the faces varied substantially within each emotion category, but could still also be said to represent the category. Difficulty score refers to the level of ability required to correctly identify an expression in the image, ranging from positive to negative. When scores are more negative, most individuals can correctly identify the expression in that image, most of the time, because these expressions are “easy” in the sense that relatively low levels of ability in identifying expressions of the type in question (happy, fear, etc.) are required in order to correctly identify them. In contrast, more positive scores indicate that relatively few individuals will correctly identify the expression in the image provided, most of the time, because higher levels of ability in identifying expressions of the type in question (happy, fear, etc.) are required in order to correctly identify them. Furthermore, the in-fit and out-fit mean square statistics were used to narrow down the faces in the CAFE Subset B. The in-fit is an index of unexpected responses to items that have a difficulty score (bi) that is close to an individual’s ability (e.g., cases where an individual responds incorrectly to an item that is easy with respect to his/her ability). The out-fit is an index of unexpected responses to items that have a difficulty score (bi) that is far from an individual’s ability (e.g., cases where an individual responds correctly to an item that is too difficult for his/her ability). In-fit and outfit scores lower than 0.5 indicate a lack of reliability, whereas in-fit and out-fit scores greater than 1.5 indicate noise. Thus, Subset B is comprised of the faces that fit within the 0.5–1.5 range. Thus, faces selected for Subset B were reliable but had varying degrees of difficulty. Importantly, the difficulty scores (*b*_*i*_) were highly correlated with the accuracy scores, *r* = −0.858, *p* < 0.001, so we used accuracy scores for each item in CAFE Subset B to construct our smaller subsets, since they are easier to interpret than difficulty scores (see LoBue and Thrasher, 2015, for full description of these analyses).

##### Face selection plan

We planned to identify a subset of 20 faces for each emotion category in CAFE Subset B that have accuracy scores that follow a normal distribution, so that the degree to which each face is easy or difficult to identify is standardized, and thus optimized to capture variability in a sample of observers. Ideally, we could construct a normal distribution with the empirically derived mean and standard deviation of the accuracy scores for each emotion category in CAFE Subset B, and then identify 20 face images with accuracy scores corresponding to the 2.5th, 7.5th, …, 97.5th percentiles in the cumulative distribution function (CDF) of the constructed normal distribution for each emotion category. However, we found that the distributions of accuracy scores for some emotion categories in CAFE were slightly left skewed, with mean accuracy scores above 0.5 (50%), so the range of the 20 percentile values of the constructed normal distribution was beyond the 0–1 range. For example, in the constructed normal distribution for angry faces, the 97.5th percentile was 1.09. However, there are no angry face exemplars with an accuracy score of 1.09, or 109% correct. Thus, we could not select faces that correspond to 2.5–97.5th percentiles of the CDF for all emotion categories. Furthermore, in the pool of angry faces, the highest accuracy score reported in LoBue and Thrasher (2015) was 0.95 (or 95%) which only corresponded to the 90th percentile of the constructed normal distribution.

Several alternative strategies are possible. First, we could construct the normal distribution with a smaller mean than the one reported in LoBue and Thrasher (2015), to move the 20 percentile values in the constructed normal distribution within the 0–1 range. However, using this method would result in having a different mean of accuracy scores than collected in the original validation of the CAFE set. Another possibility is to construct the normal distribution with a smaller standard deviation to make the range of the 20 percentile values in the constructed normal distribution within 0–1. However, this method would result in a smaller variance than that observed in the original CAFE set. Alternatively, we could choose faces from a smaller range of percentiles (e.g., 10–90th percentiles) of the normal distribution, resulting in the same mean and standard deviation as in the original set, but with short tails for each distribution. With this method, we would have normal distributions of accuracy scores, but we may lose the meaningful skew represented in the original distribution. One final option is to select 20 faces from the full set based on its natural semi-normal distribution. Using this method, we would have the same natural distributions of accuracy scores as in the original face set, which could be meaningful for some research that aims to examine participants’ responses to naturally occurring distributions of difficulty among emotional expressions, although they may not be perfectly normal.

We decided to construct two subsets of faces, using both latter two methods. We chose this strategy so that we could have one subset that maintains a similar distribution to the original set, and another subset that has a normal distribution of accuracy scores for each emotion category. Thus, here we will create, pilot, and validate two subsets of the original CAFE set both containing 140 faces with a variety of difficulty levels for researchers to choose from: CAFE-S1, which has a standardized distribution (normal distribution) of accuracy scores for each emotion category, and CAFE-S2, which mimics the natural distributions of accuracy scores in the in CAFE Subset B.

##### Face selection for CAFE-S1

For CAFE-S1, we used adults’ accuracy scores from LoBue and Thrasher (2015) to identify a subset of 20 faces for each of the seven emotion categories that have accuracy scores following a normal distribution, and the same means and standard deviations as in the full CAFE Subset B. We did this in three steps. First, we calculated the mean and standard deviation of all accuracy scores of the faces for each emotion category based on the ratings reported in LoBue and Thrasher (2015). Next, we constructed a normal distribution with these empirically derived means and standard deviations. Finally, we identified the 20 evenly distributed percentiles of the constructed normal distributions and looked for 20 faces with accuracy scores that matched (or were closest to) these percentiles.

For emotion categories with accuracy scores that covered the full range of 2.5–97.5th percentile of the distribution, we chose the 20 evenly distributed percentiles that cover the whole distribution (i.e., the 2.5th, 7.5th, 12.5th, …, 97.5th percentiles), and identified faces with matching accuracy scores. For emotion categories with accuracy scores that did not reach at least one end of the range (e.g., when the distribution was skewed, with accuracy scores that only ranged from the 1st percentile to the 88th percentile), we chose the 20 evenly distributed percentiles that centered around the median and covered the largest range where data were available (in the example above, the 12th, 16th, 20th, …, 88th percentiles), and identified matching faces.

##### Face selection for CAFE-S2

For CAFE-S2, we identified another subset of 20 faces for each emotion category that have the same natural distribution of accuracy scores as CAFE Subset B, using adults’ accuracy scores from LoBue and Thrasher (2015). For each emotion category, we first sorted the faces based on the accuracy scores reported in LoBue and Thrasher (2015), and then selected 20 faces with an equal interval, *k*, and an initial order number, *k/2 (k = N/n*), where *N* is the number of faces in the CAFE Subset B for the particular emotion category and *n* is the number of faces we aimed to select (20). For example, if we were to choose 20 faces from 100 angry faces, we would choose the 3rd, 8th, 13th, 18th,…and 98th angry faces from the CAFE Subset B.

At the same time, for both subsets, we attempted to include half male and half female faces, retain the same composition of race/ethnicity of the faces as in the full set, and achieve a balance in accuracy scores among different races/ethnicities and genders of the faces. If there was more than one face that matched or was close to the target percentile, we selected the face that was most helpful for balancing the accuracy scores across gender and race/ethnicity.

Notably, in both subsets, we retained the variability in the mean accuracies across emotions (e.g., relatively high mean accuracy for happy faces and relatively low mean accuracy for fearful faces), as this variability is consistent with other studies on emotion recognition and may reflect natural and meaningful variability in human’s ability to recognize or display different emotions.

### Results

#### CAFE-S1

Demographic information for our selected subset of 140 faces is listed in Table 1. It consists of 67 male and 73 female faces, and the composition of ethnicity/race of the faces mirrors that of the models in the entire CAFE set. Shapiro–Wilk tests of normality for the accuracy distributions of the selected faces in all seven emotion categories were not significant, indicating the distributions for the 20 faces selected for each emotion category were normal. The histograms and Q-Q plots of the distributions of the 20 selected faces for each emotion in CAFE-S1 were presented in Supplementary Materials (see Supplementary Figures S1, S4), together with the histograms of the accuracy scores for faces in the original CAFE Subset B (see Supplementary Figure S3).

**TABLE 1:** The demographic information of the faces selected in CAFE-S1.

		Angry	Happy	Sad	Surprise	Neutral	Disgust	Fear	Total	Percentage in CAFE-S1	Percentage in the full CAFE Set^*a*^	Percentage of all child models in the full CAFE Set
Gender	Male	10	10	8	11	10	9	9	67	47.86	37.80	41.56
	Female	10	10	12	9	10	11	11	73	52.14	62.20	58.44
Mouth	Open	10	11	10	20	10	12	10	83	59.29	51.10	
	Close	10	9	10	0	10	8	10	57	40.71	48.90	
Ethnicity	AA	3	3	4	3	4	3	4	24	17.14	20.64	17.53
	White	10	10	8	11	10	10	10	69	49.29	43.39	50.00
	EA	2	2	2	2	2	2	2	14	10.00	11.47	10.39
	SA	2	2	3	1	1	2	1	12	8.57	9.54	7.14
	LA	3	3	3	3	3	3	3	21	15.00	14.95	14.93
Total		20	20	20	20	20	20	20	140	100		

*^*a.*^This column indicates the percentage in the full CAFE set. For example, the first number 37.80 indicates the percentage of all male photos in the full CAFE set is 37.80%. AA, African American; White, White, non-Hispanic or Latino; EA, East Asian; SA, South Asian; LA, Latino or Hispanic American.*

##### Angry

CAFE Subset B contains 197 images of children displaying angry facial configurations with mouths either open or closed. The distribution of the mean ratings of all 197 angry faces was left skewed with a mean of 0.67 and a standard deviation of 0.21. In a normal distribution with a mean of 0.67 and a standard deviation of 0.21, the highest available accuracy score for all angry facial configurations was at the 90.14th percentile. Therefore, we selected 20 facial configurations with accuracy scores closest to the 10th, 14.2th, 18.4th,…, 90th percentile of the normal distribution with a mean of 0.67 and a standard deviation of 0.21.

##### Disgust

CAFE Subset B contains 182 faces of children displaying disgusted facial configurations. The mean rating was 0.65 with a standard deviation of 0.18. In the normal distribution with a mean of 0.65 and a standard deviation of 0.18, the accuracy scores range from the 0.19th to the 95.15th percentile. We selected the 20 disgusted facial configurations closest to the 2.5th, 7.5th,…, 97.5th percentile with an increment of 5 percentiles.

##### Fear

CAFE Subset B contains 136 pictures of children displaying fearful facial configurations. We constructed a normal distribution with the mean rating score of 0.43, and a standard deviation of 0.18. The percentiles for the lowest and the highest accuracy score of fearful configurations were at the 1.26th and 98.50th percentile, respectively. Therefore, we selected the 20 fearful configurations with ratings corresponding to the 2.5th to the 97.5th of the normal distribution with an increment of 5 percentiles.

##### Happy

The mean of the 172 happy configurations in CAFE Subset B was 0.83, with a standard deviation of 0.17. Because the distribution of these 172 happy faces was left skewed, the highest score of the happy facial configurations was at the 80.64th percentile of the constructed normal distribution. We therefore selected 20 happy facial configurations with ratings from the 20th to the 80th percentile with an increment of 3.16 percentiles.

##### Neutral

The mean rating of the 194 configurations in CAFE Subset B was 0.65, with a standard deviation of 0.27. The lowest score was corresponding to 0.92th percentile and the highest score was corresponding to the 87.99th percentile of the normal distribution with a mean of 0.65 and a standard deviation of 0.27. We selected 20 facial configurations with ratings closest to the 10th, 14.2th, …, 90th percentile of the normal distribution with an increment of 4.2 percentiles.

##### Sad

The distribution of the 106 sad configurations in CAFE Subset B was also slightly left skewed, with a mean of 0.62, and a standard deviation of 0.23. The lowest and the highest score of all sad facial configurations were corresponding to 0.95th and 92.61th percentile in the constructed normal distribution. Twenty sad facial configurations with accuracy scores closest to the 7.5th, 11.97th, …, 92.5th with an increment of 4.47 percentiles were selected.

##### Surprise

There were 103 surprised facial configurations in CAFE Subset B, with a mean rating of 0.72 and a standard deviation of 0.12. The lowest score and the highest score of the facial configurations were at the 1.86th and 98.10th percentile of the normal distribution with a mean of 0.72 and a standard deviation of 0.12. We selected 20 surprised facial configurations which corresponded to the 2.5th, 7.5th, …, 97.5th percentile of the normal distribution with an increment of 5 percentiles.

##### Analysis of CAFE-S1

Using the accuracy scores reported in LoBue and Thrasher (2015), we conducted a 2 (face gender) × 5 (face race/ethnicity) × 7 (emotion) analysis of variance (ANOVA) to examine whether there were any significant differences in the accuracy scores of selected facial configurations among genders, races/ethnicities, and emotion categories. There was only a significant effect of emotion category, *F*(6,128) = 10.55, *p* < 0.001, with a highest mean accuracy score for happy facial configurations, and a lowest mean accuracy score for fearful facial configurations, as in the CAFE Subset B. The effects of gender, *F*(1,128) = .39, *p* = 0.54, and face race *F*(4,128) = 0.98, *p* = 0.42, were not significant, meaning there was no significant difference between faces of males and females, or among different ethnicities/races of the faces.

#### CAFE-S2

Demographic information for the selected 140 faces in CAFE-S2 is listed in Table 2. It contains 70 male and 70 female faces, and the ethnicity/race mirrors the composition in the full set. ANOVA showed no significant effect of face gender, *F*(1,128) = 1.66, *p* = 0.20, or face race, *F*(4,128) = 0.98, *p* = 0.42, but a significant effect of face emotion on the accuracy scores reported in LoBue and Thrasher (2015), *F*(6,128) = 6.98, *p* < 0.001. The mean accuracy score of happy facial configurations was higher than other faces, and that of fearful facial configurations was lower than other faces, as in the CAFE Subset B. The histograms of the distributions of the 20 selected faces for each emotion in CAFE-S2 were presented in Supplementary Materials (see Supplementary Figure S2).

**TABLE 2:** The demographic information of the faces selected in CAFE-S2.

		Angry	Happy	Sad	Surprise	Neutral	Disgust	Fear	Total	Percentage in CAFE-S2	Percentage in the full CAFE Set^*a.*^	Percentage of all child models in the full CAFE Set
Gender	Male	9	12	8	10	11	10	10	70	50.00	37.80	41.56
	Female	11	8	12	10	9	10	10	70	50.00	62.20	58.44
Mouth	Open	8	9	7	20	9	10	9	72	51.43	51.10	
	Close	12	11	13	0	11	10	11	68	48.57	48.90	
Ethnicity	AA	4	3	4	4	4	3	3	25	17.86	20.64	17.53
	White	9	10	9	9	9	10	10	66	47.14	43.39	50.00
	EA	2	2	2	2	2	2	2	14	10.00	11.47	10.39
	SA	2	2	2	2	2	2	2	14	10.00	9.54	7.14
	LA	3	3	3	3	3	3	3	21	15.00	14.95	14.93
Total		20	20	20	20	20	20	20	140	100		

*^*a*^This column indicates the percentage in the full CAFE set. For example, the first number 37.80 indicates the percentage of all male photos in the full CAFE set is 37.80%. AA, African American; White, White, non-Hispanic or Latino; EA, East Asian; SA, South Asian; LA, Latino or Hispanic American.*

### Discussion

In Study 1, we constructed two subsets of 140 faces from CAFE Subset B. Each small subset contains 20 faces from each of the six basic emotion categories—happy, sad, fearful, disgusted, surprised, angry—and 20 neutral facial configurations. In CAFE-S1, the 20 faces for each emotion category formed a normal distribution of accuracy scores, whereas, in CAFE-S2, the 20 faces for each emotion category followed the same distribution as the full CAFE set.

## Study 2

The aim of Study 2 was to pilot test the selected faces with 100 new adult participants for each subset. We then examined the distribution of newly collected accuracy scores for the stimuli in CAFE-S1 and checked whether these accuracy scores were well-balanced between genders and races/ethnicities in both small subsets, so that adjustments could be made accordingly.

### Method

One hundred adults (49 male, 50 female, one preferred not to indicate gender) identified the emotion categories represented in the 140 photos in CAFE-S1 (Survey 1) on Mechanical Turk. The sample size was based on previous studies using similar methods (Tottenham et al., 2009; LoBue and Thrasher, 2015; Moyal et al., 2018). The sample was 12% African American, 5% East Asian, 70% White, 1% South Asian, 4% Latino, 7% mixed, and 1% did not indicate their race/ethnicity. Another 100 (50 male, 49 female, one preferred not to indicate gender) adults identified the 140 photos in CAFE-S2 (Survey 2) on Mechanical Turk. The sample was 7% African American, 5% East Asian, 74% White, 1% South Asian, 3% Latino, 5% mixed, 3% Middle Eastern, 2% Native American.

After giving consent, participants were asked to provide demographic information, such as gender, age, and race/ethnicity. They were then presented with the 140 faces in CAFE-S1 (Survey 1) or CAFE-S2 (Survey 2), and an additional seven emoji cartoon pictures representing the seven emotion categories contained within each subset. The seven emoji pictures were used as an attention check to ensure that participants were attending to the task, and participants’ data were eliminated if they failed to accurately identify three or more of the emojis. On each successive trial, a single face appeared on the screen and the participant was prompted to choose whether the face was happy, sad, surprised, angry, disgusted, fearful, or neutral. The face remained on the screen until the participant clicked on a response and continue to the next face. The same method was used in LoBue and Thrasher (2015). All 147 pictures were presented in a random order. The study lasted approximately 12 min and each participant received 75 cents in compensation. Data were collected from an additional six participants for Survey 1, and additional 23 participants for Survey 2, but were excluded for failure to pass the attention check.

### Results

#### Survey 1 (CAFE-S1)

The means and standard deviations of the accuracy scores collected in Study 2 for each emotion category are listed in Table 3. Shapiro–Wilk tests showed that the ratings of the 20 faces for each emotion category were all normally distributed, *p*s > 0.05. The correlation between the ratings for CAFE-S1 and the ratings reported in LoBue and Thrasher (2015) was *r* = 0.88, *p* < 0.001. A 2 (gender) × 5(face race) × 7 (emotion category) ANOVA, with the main effects and the interaction effects of emotion with face gender and face race, on face level ratings collected in Study 2 showed that there was no significant difference in ratings between male faces and female faces, *F*(1,98) = 3.14, *p* = 0.08, or among the five different races/ethnicities of faces, *F*(4,98) = 0.3941, *p* = 0.80. The effect of emotion category was significant, *F*(6,98) = 4.84, *p* < 0.001, again, with highest ratings for happy facial configurations, and lowest ratings for fearful facial configurations. There was no significant interaction effect between emotion with face gender, *F*(6,98) = 1.31, *p* = 0.26, or face race, *F*(24,98) = 0.94, *p* = 0.55.

**TABLE 3:** The means and standard deviations of the ratings for the selected 20 faces for each emotion (ratings from previous study LoBue and Thrasher, 2015, and ratings from our Study 2 and Study 3).

	Angry	Happy	Sad	Surprise	Neutral	Disgust	Fear
Accuracies in LoBue and Thrasher (2015)	0.67 (0.21)	0.83 (0.17)	0.63 (0.23)	0.72 (0.12)	0.65 (0.27)	0.65 (0.18)	0.43 (0.18)
*Study 2*							
Accuracies in Survey 1 (CAFE-S1)	0.64 (0.16)	0.90 (0.08)	0.63 (0.24)	0.78 (0.15)	0.61 (0.25)	0.72 (0.18)	0.48 (0.25)
Accuracies in Survey 2 (CAFE-S2)	0.64 (0.21)	0.90 (0.14)	0.62 (0.30)	0.73 (0.17)	0.60 (0.30)	0.66 (0.17)	0.47 (0.21)
*Study 3*							
Accuracies in Study 3 (CAFE-S1)	0.63 (0.14)	0.89 (0.09)	0.55 (0.23)	0.74 (0.16)	0.47 (0.27)	0.63 (0.22)	0.53 (0.25)
Accuracies in Study 3 (CAFE-S2)	0.66 (0.23)	0.89 (0.15)	0.59 (0.32)	0.74 (0.18)	0.60 (0.33)	0.67 (0.18)	0.47 (0.24)

#### Survey 2 (CAFE-S2)

The means and standard deviations of the accuracy scores for each emotion category are listed in Table 3. The correlation between ratings for CAFE-S2 and the ratings reported in LoBue and Thrasher (2015) was *r* = 0.93, *p* < 0.001. ANOVA on face level means showed no significant effect of face race, *F*(4,98) = 0.97, *p* = 0.43, or face gender, *F*(1,98) = 0.80, *p* = 0.37. Again, there was a significant effect of emotion category on the ratings, *F*(6,98) = 4.65, *p* < 0.001, with the highest for happy facial configurations and lowest for fearful facial configurations. There was no significant interaction effect for emotion with face race, *F*(24,98) = 0.89, *p* = 0.61, or face gender, *F*(6,98) = 1.50, *p* = 0.19.

### Discussion

In sum, the accuracy scores collected in Study 2 for our new small subsets of children’s emotional facial configurations were highly correlated with the ratings in LoBue and Thrasher (2015) for CAFE Subset B. The distributions of accuracy scores in CAFE-S1 were normal, and the accuracy scores were well balanced across face genders and races/ethnicities in both subsets. From this pilot study, we concluded that no necessary changes were needed for the two new subsets. Therefore, without adjustment, we further validated the same two subsets together with a larger sample in Study 3.

## Study 3

### Methods

One thousand adults (491 males, 499 females, 10 indicated other or preferred not to indicate gender) participated in the study on Mechanical Turk. The sample was 8.8% African American, 5.7% East Asian, 72.7% White, 0.9% South Asian, 3.9% Latino, 1.1% Native American, 0.2% Pacific Islander, 5.7% mixed, and 1% did not indicate their race/ethnicity. Although the majority of raters in this study and the previous study were White, the original raters in LoBue and Thrasher (2015) were quite diverse (17% African American, 27% Asian, 30% White, and 17% Latino, 9% chose “Other” or did not indicate their race/ethnicity), and the current ratings revealed nearly identical patterns to those.

The procedure was nearly identical to that of Study 2, except that participants were presented with all 235 faces (among these 95 in CAFE-S1 only, 95 in CAFE-S2 only, and 45 in both CAFE-S1 and CAFE-S2), and an additional seven emoji cartoon pictures. For each trial, participants were again presented with a single photo, and were asked to choose from happy, sad, surprised, angry, disgusted, fearful, or neutral to indicate the best emotion category to describe each facial configuration. The study lasted approximately 25 min and each participant received $3 for compensation. An additional 56 participants completed the survey but were excluded from the study for failure to pass the attention check.

### Results

With the ratings from the 1000 participants, we calculated the mean accuracy for each face and analyzed the accuracy scores for faces in CAFE-S1 and CAFE-S2 separately. The means and standard deviations of the accuracy scores for each emotion category are presented in Table 3.

#### CAFE-S1

Validity scores were indexed by the percentage of the 1000 participants to correctly categorize the photographs, as in LoBue and Thrasher (2015). There was substantial variability in accuracy scores across the 140 faces of the subset, with a mean of 0.64 and a range of 0.10–0.99. The mean ratings for each face in Study 3 were highly correlated with the ratings in Study 2, *r* = 0.92, *p* < 0.001, and the ratings reported in LoBue and Thrasher (2015), *r* = 0.84, *p* < 0.001. Paired *t*-test showed no significant difference between the ratings collected in Study 3 and LoBue and Thrasher (2015), *t* = 1.53, *p* = 0.13. Shapiro–Wilk tests showed that the ratings of the 20 faces for each emotion category in CAFE-S1 were all normally distributed, *p*s > 0.05. Further, we conducted 2 (face gender) × 5 (face race/ethnicity) × 7 (emotion category) ANOVA to examine the main and interaction effects of face gender, face race/ethnicity, and emotion category on the ratings. There was only a significant main effect of emotion category, *F*(6,95) = 5.20, *p* < 0.001. The main effect of face gender, *F*(1,95) = 1.90, *p* = 0.17, face race, *F*(4,95) = 1.02, *p* = 0.40, and the interaction effects, *F* = 0.87–1.20, *p* = 0.31–0.52, were not significant.

#### CAFE-S2

As for CAFE-S1, validity scores for CAFE-S2 were obtained by calculating the accuracy scores for each photograph among the 1000 participants who completed Survey 3. The accuracy scores had a mean of 0.66, and a range of 0.04–0.99 (see Table 3 for the means and standard deviations of the accuracy scores for each emotion category). In addition, there were strong correlations between the mean ratings in Study 3 and Study 2, *r* = 0.98, *p* < 0.001, and between Study 3 and LoBue and Thrasher (2015), *r* = 0.93, *p* < 0.001. Paired *t*-test showed no significant differences between the ratings between Study 3 and LoBue and Thrasher (2015), *t* = −0.48, *p* = 0.64. Further, an ANOVA again revealed a significant main effect of emotion category, *F*(6,95) = 4.53, *p* < 0.001. The main effect of face gender, *F*(1,95) = 0.74, *p* = 0.39, face race, *F*(4,95) = 0.78, *p* = 0.54, and the interaction effects, *F* = 0.92–1.81, *p* = 0.15–0.37, were not significant.

## General Discussion

The aim of the current study was to develop a small subset of the CAFE to be used in future research with children. The result of this investigation was two validated subsets that will enable researchers to study individual differences in children’s emotion recognition with a highly diverse and variable set of children posing for 6 basic emotions and a neutral expression. We will provide two subsets for researchers to choose from: CAFE-S1 is made up of 140 exemplars, and has accuracy scores that fall on a normal distribution for each emotion category; CAFE-S2 is also made up of 140 exemplars, and mimics the natural distribution of accuracy scores in the full CAFE Subset B. A full list of the faces in each subset along with the validation collected here is available on Databrary^2^.

Although there are countless stimulus sets with photographs of posed emotion configurations available online, most only contain depictions of Caucasian adults’ stereotyped emotion configurations, with limited diversity in face race or ethnicity and levels of intensity or difficulty. The two newly constructed subsets of CAFE are highly diverse, and are designed to produce variability in naïve observers, minimizing the possibility of ceiling effects that are often likely with stimulus sets only containing highly iconic, stereotyped displays. Further, especially for studies with children, the stimulus size of 140 exemplars will help researchers seeking to use faces in CAFE choose from a more manageable sized stimulus. The subsets have been validated with a large sample to ensure that the selected stimuli are well balanced in race/ethnicity and gender, which is important for researchers seeking to study group differences.

As with any stimulus set, our subsets carry with them various limitations. They are of course made up of static images of children posing for a limited number of emotion configurations. Indeed, emotions are dynamic, multi-modal systems, that elicit not only changes in facial affect, but also in the body and in the voice. Our subsets, like the full CAFE set, do not include dynamic stimuli, where bodily and vocal information is also available for each emotion category. Further, although our subsets feature variability in iconicity, they are still posed configurations, which may lack the variability contained within more natural, spontaneous facial expressions. Despite these limitations, our subsets are still incredibly valuable tools for psychological researchers. Indeed, CAFE is the *single most downloaded library* on Databrary, and is currently being used by over 90 researchers and clinicians from around the world for basic science and as an assessment tool for children at risk for various developmental disabilities. Further, although the set has only been available for less than 5 years, it has already been used or cited in over 100 publications. Thus, the small subsets have the potential of making CAFE use even easier and more widespread.

In conclusion, here we present and validate two new subsets of photographs of children’s facial configurations for researchers to use in emotion research. These two subsets will contribute to the field significantly by providing researchers with an important tool for the investigation of children’s emotion perception.

## Data Availability Statement

The raw data supporting the conclusions of this article will be made available by the authors, without undue reservation.

## Ethics Statement

The studies involving human participants were reviewed and approved by the Rutgers University Institutional Review Board for the Protection of Human Subjects. The patients/participants provided their written informed consent to participate in this study. Written informed consent was obtained from the minor(s)’ legal guardian/next of kin for the publication of any potentially identifiable images or data included in this article.

## Author Contributions

YY and VL developed the subsets of photographs and wrote the manuscript. YY collected and analyzed the data. Both authors contributed to the article and approved the submitted version.

## Conflict of Interest

The authors declare that the research was conducted in the absence of any commercial or financial relationships that could be construed as a potential conflict of interest.

## Footnotes

1 This study aimed to select faces from CAFE Subset B to develop subsets of faces with a variety of difficulty levels to recognize. For researchers who prefer to use stereotypical exemplars only, they can use CAFE Subset A to obtain a small sample of the most stereotypical depiction in each category, e.g., by choosing 10 or 20 faces from each emotion category that have the highest accuracy scores in CAFE Subset A.

2 https://nyu.databrary.org/volume/1170

3 http://databrary.org/volume/30

## References

[B1] Barrett L. F. Adolphs R. Marsella S. Martinez A. M. Pollak S. D. (2019). Emotional expressions reconsidered: challenges to inferring emotion from human facial movements. *Psychol. Sci. Public Interest* 20 1–68. 10.1177/1529100619832930 31313636 PMC6640856

[B2] Ekman P. Friesen W. V. (1971). Constants across cultures in the face and emotion. *J. Pers. Soc. Psychol.* 17 124–129. 10.1037/h0030377 5542557

[B3] Elfenbein H. A. Ambady N. (2002). Is there an in-group advantage in emotion recognition? *Psychol. Bull.* 128 243–249. 10.1037/0033-2909.128.2.243 11931518

[B4] Gao X. Maurer D. (2010). A happy story: developmental changes in children’s sensitivity to facial expressions of varying intensities. *J. Exp. Child Psychol.* 107 67–86. 10.1016/j.jecp.2010.05.003 20542282

[B5] Ishii K. Reyes J. A. Kitayama S. (2003). Spontaneous attention to word content versus emotional tone: differences among three cultures. *Psychol. Sci.* 14 39–46. 10.1111/1467-9280.01416 12564752

[B6] Jack R. E. Garrod O. G. B. Yu H. Caldara R. Schyns P. G. (2012). Facial expressions of emotion are not culturally universal. *Proc. Natl. Acad. Sci. U.S.A.* 109 7241–7244. 10.1073/pnas.1200155109 22509011 PMC3358835

[B7] LoBue V. Thrasher C. (2015). The child affective facial expression (CAFE) set: validity and reliability from untrained adults. *Front. Psychol.* 5:1532. 10.3389/fpsyg.2014.01532 25610415 PMC4285011

[B8] Ma D. S. Correll J. Wittenbrink B. (2015). The Chicago face database: a free stimulus set of faces and norming data. *Behav. Res. Methods* 47 1122–1135. 10.3758/s13428-014-0532-5 25582810

[B9] Moyal N. Henik A. Anholt G. E. (2018). Categorized affective pictures database (CAP-D). *J. Cogn.* 1:41. 10.5334/joc.47 31517214 PMC6634429

[B10] Tanaka A. Koizumi A. Imai H. Hiramatsu S. Hiramoto E. de Gelder B. (2010). I feel your voice: cultural differences in the multisensory perception of emotion. *Psychol. Sci.* 21 1259–1262. 10.1177/0956797610380698 20713633

[B11] Tottenham N. Tanaka J. W. Leon A. C. McCarry T. Nurse M. Hare T. A. (2009). The NimStim set of facial expressions: judgments from untrained research participants. *Psychiatry Res.* 168 242–249. 10.1016/j.psychres.2008.05.006 19564050 PMC3474329

