# Supplementary material for: The Child Affective Facial Expression Set Short Versions (CAFE-Ss): Development and Validation of Two Subsets of Children’s Emotional Faces With Variability
Source: Front Psychol. 2020 Dec 4;11:599245. doi: 10.3389/fpsyg.2020.599245 (PMC7746875; doi:10.3389/fpsyg.2020.599245)

Figure S1. The histograms of the accuracy scores for selected faces in CAFE-S1 (ratings in LoBue & Thrasher, 2015)

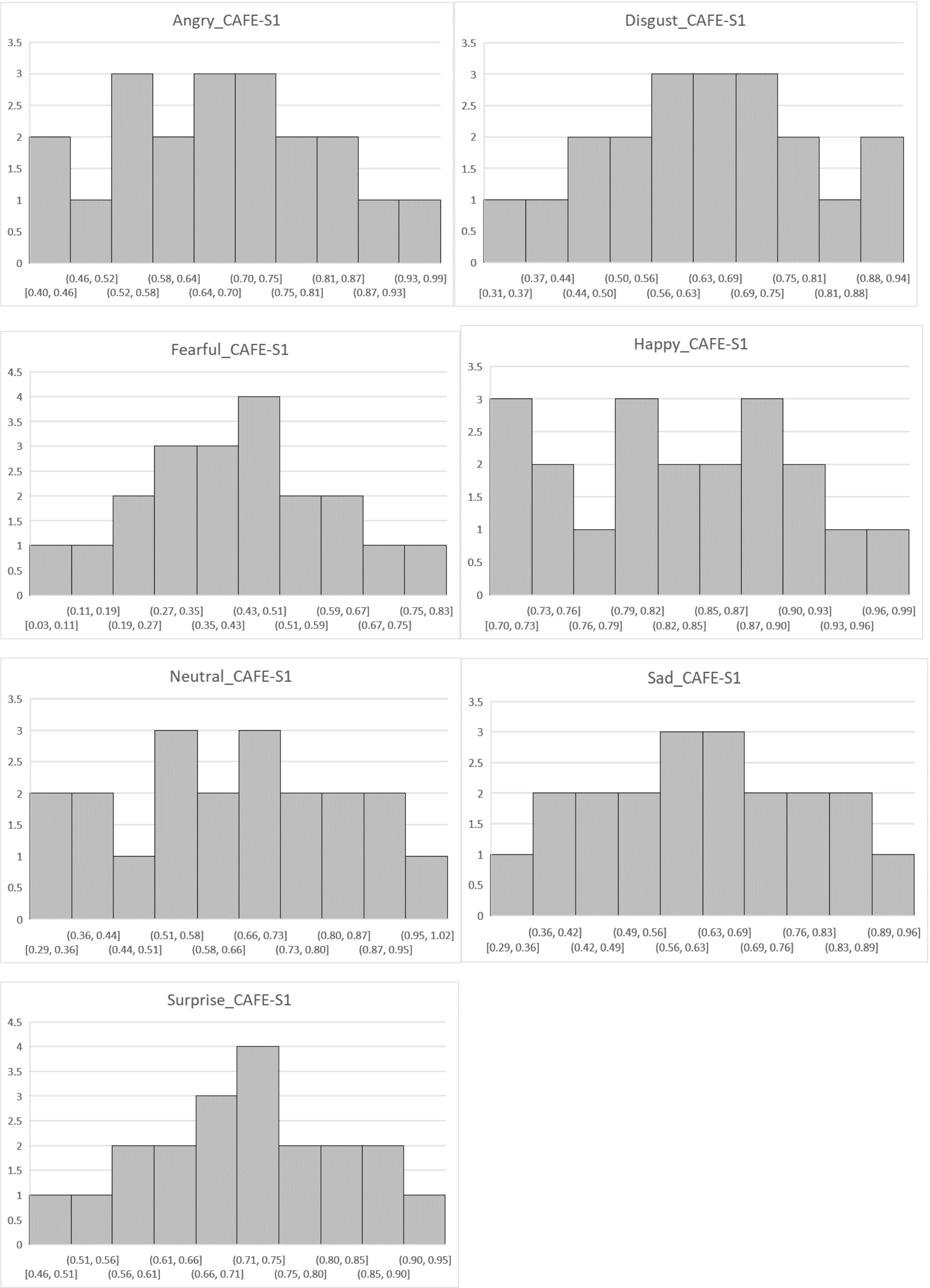

Figure S2. The histograms of the accuracy scores for selected faces in CAFE-S2 (ratings in LoBue & Thrasher, 2015)

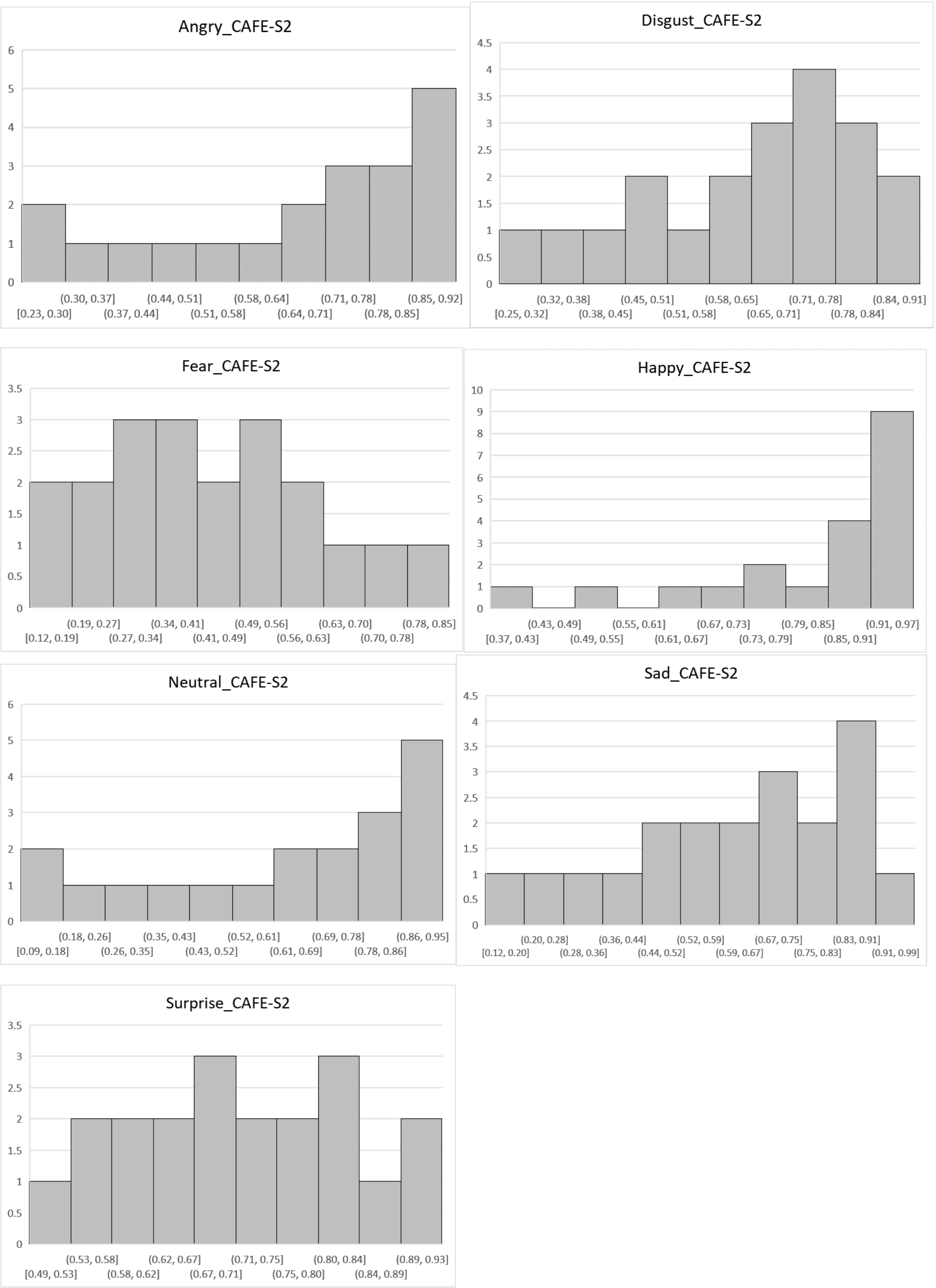

Figure S3. The histograms of the accuracy scores for faces in the original CAFE Subset B (ratings in LoBue & Thrasher, 2015)

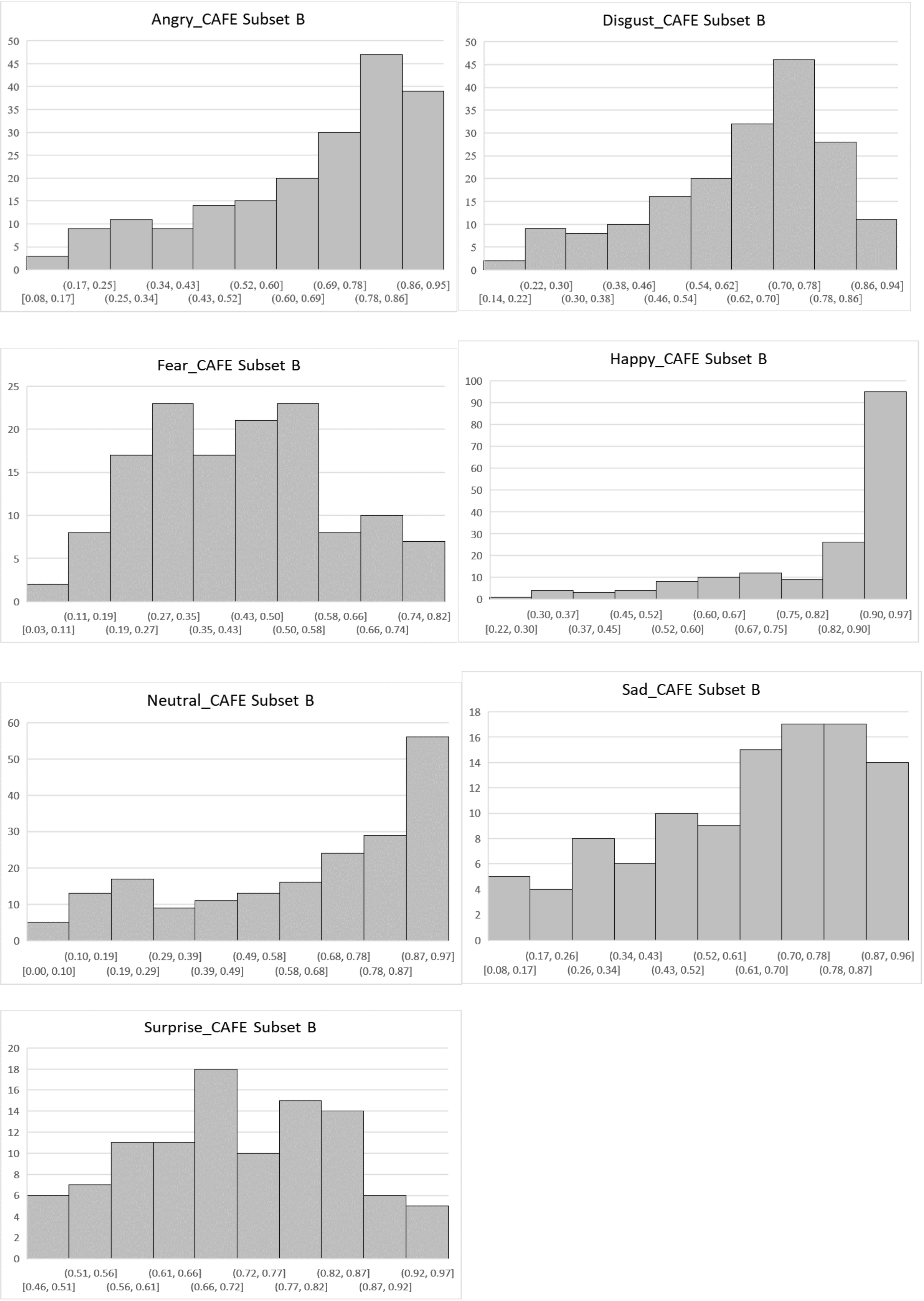

Figure S4. The Q-Q plots of the distributions of the 20 selected faces for each emotion in CAFE-S1 (ratings in LoBue & Thrasher, 2015).

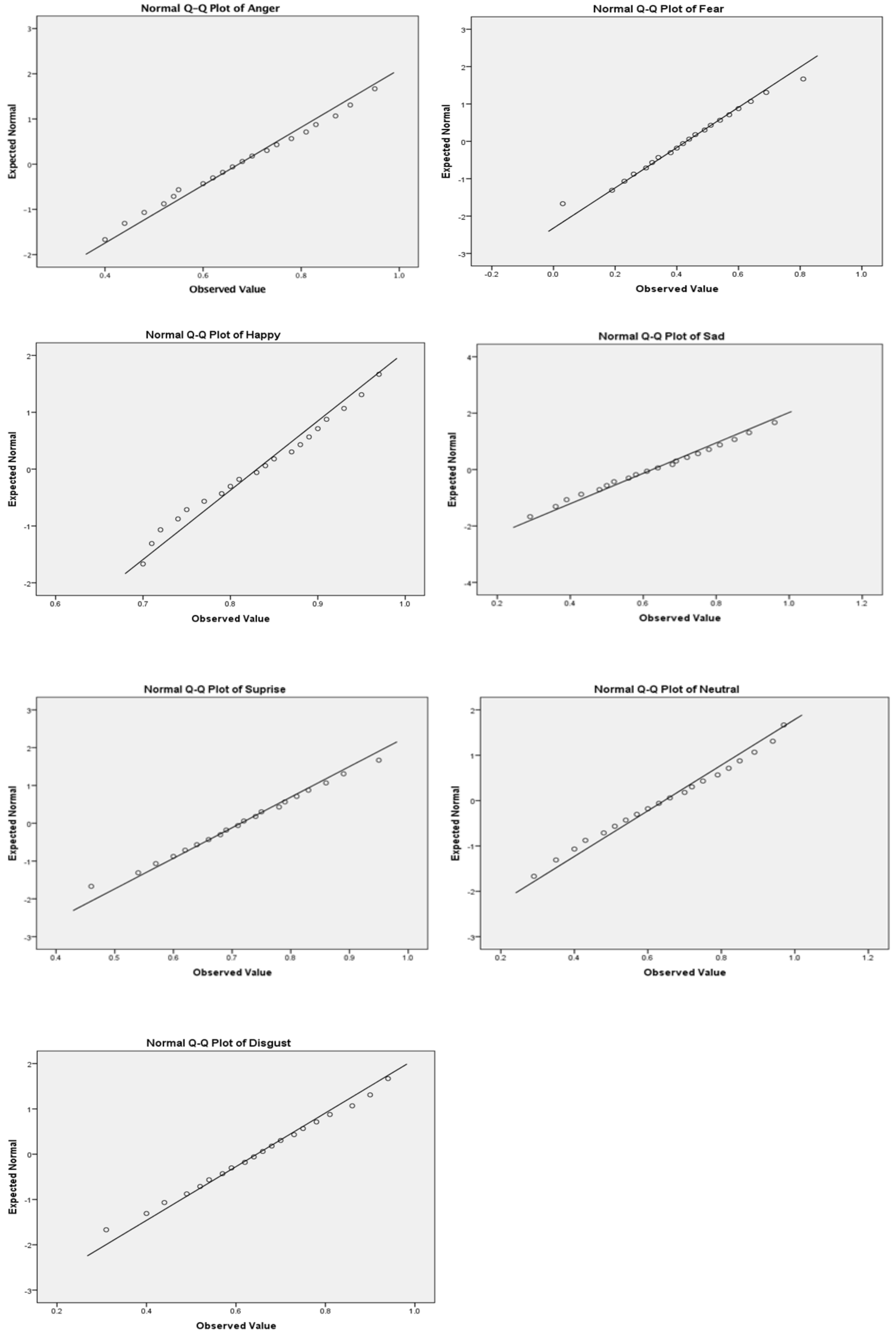

Supplement: Supplementary file 1 [file Image_1.pdf]
